# Distinct Associations of *GTF2I*, *TP53*, and *NOTCH1* Variants with Indolent and Aggressive Thymic Epithelial Tumors in Vietnamese Patients

**DOI:** 10.3390/genes17050524

**Published:** 2026-04-29

**Authors:** Duc Manh Le, Thi Xuan Nguyen, Thanh Chung Dang, Ngoc Dung Tran, Ngoc Lan Nguyen, Thai Tra Dang, Thu Hien Nguyen, Huy Hoang Nguyen, Duc Quan Nguyen, Thi Trang Do

**Affiliations:** 1 Department of Pathology and Forensic Medicine, Military Hospital 103, Vietnam Military Medical University, Ha Dong, Hanoi 261000, Vietnam; manhld1982@gmail.com (D.M.L.); dangthanhchung@vmmu.edu.vn (T.C.D.); tranngocdung_gpb@vmmu.edu.vn (N.D.T.); dangthaitra0502hvqy@gmail.com (T.T.D.); thuhienth87@gmail.com (T.H.N.); 2 Research Centre 69, Ho Chi Minh President Mausoleum Command, Ba Dinh, Hanoi 10000, Vietnam; 3 Institute of Biology, Vietnam Academy of Science and Technology, Nghia Do, Hanoi 10000, Vietnam; xtltam76@gmail.com (T.X.N.); hoangibt@yahoo.com (H.H.N.); ducquan0709@gmail.com (D.Q.N.); 4 Center for Gene and Protein Research, Hanoi Medical University, Kim Lien, Hanoi 11521, Vietnam; nguyenngoclan@hmu.edu.vn; 5 Publishing House for Science and Technology, Vietnam Academy of Science and Technology, Nghia Do, Hanoi 10000, Vietnam

**Keywords:** thymic epithelial tumors, *GTF2I*, *TP53*, *NOTCH1*, genetic variants, molecular marker

## Abstract

Background: Thymic epithelial tumors (TETs) are rare neoplasms of the anterior mediastinum, ranging from indolent thymomas to aggressive thymic carcinomas. Increasing evidence suggests that genetic alterations contribute to their pathogenesis and biological behavior. *GTF2I*, *TP53*, and *NOTCH1* are particularly interesting among the potential genes due to their central roles in transcriptional regulation, cell-cycle control, and oncogenic signaling. Methods: In this study, 150 TET samples from Vietnamese patients were classified according to WHO guidelines and the Masaoka–Koga staging system. Genotyping was conducted on 139 high-quality samples using Sanger sequencing targeting exon 15 of *GTF2I*, exon 7 of *TP53*, and exon 34 of *NOTCH1*. The potential impact of these variants was predicted using the *in silico* MutationTaster2025 and CADD v1.7 tools. Statistical analyses were also conducted to assess associations between variants and tumor subtypes. Results: Our study identified a total of 17 variants across *GTF2I*, *TP53*, and *NOTCH1*, in which the c.1271T>A variant in the *GTF2I* hotspot, predicted to be deleterious, was identified in 14.1% of indolent thymomas and showed a significant association with this subtype group (odds ratio: 0.048, adjusted *p*-value = 0.014). In contrast, previously unreported variants in TP53 (c.772G>A) and *NOTCH1* (c.7546T>G) were also computationally predicted to be deleterious and were significantly enriched in aggressive subtypes, with ORs of 15.1 (adjusted *p*-value = 0.01) and 18.4 (adjusted *p*-value = 0.026), respectively. Conclusion: These hypothesis-generating findings suggest that variations in *GTF2I*, *TP53*, and *NOTCH1* may serve as candidate molecular markers for distinguishing thymoma subtypes and assessing patient risk. To date, this is the first targeted hotspot screening study of *GTF2I*, *TP53*, and *NOTCH1* variants in TETs within the Vietnamese population.

## 1. Introduction

Thymic epithelial tumors (TETs) are a heterogeneous group of neoplasms arising from the epithelial cells of the thymus, a primary lymphoid organ located in the anterior mediastinum [1]. The classification of TETs typically follows the guidelines established by the World Health Organization (WHO) and the system proposed by Masaoka and Koga for histological subtyping and clinical staging, respectively [2,3,4]. The WHO classifies TETs into several histologic subtypes based on the morphology of epithelial cells and the ratio of lymphocytes to epithelial cells. These include types A, AB, B1, B2, and B3 thymomas along with thymic carcinomas [2]. The Masaoka–Koga system divides TETs into four stages dependent on the infiltration of the neighboring structures or the spread through lymphatic and hematogenous routes [3,4]. Previous studies have also found that thymomas can be further divided into two groups according to their aggressiveness and recurrence rate as well as overall prognosis. Types A, AB, and B1 are classified within a group of thymomas that exhibit low-risk/less aggressive behaviors due to their indolent characteristics and favorable outcomes. In contrast, types B2 and B3 constitute a distinct group, identified as high-risk/more aggressive thymomas due to their greater invasiveness, advanced clinical stages, and less favorable prognoses [5,6]. The incidence of TETs is estimated at 0.1 to 0.3 cases per 100,000 individuals each year, with the highest occurrence observed in those aged 40 to 60, regardless of gender [1,7].

Their pathogenesis is increasingly recognized as being caused by alterations in critical molecular pathways. Many studies have investigated the underlying causes of TETs at the molecular level, focusing on mutations in various key transcription factors and functional genes across diverse populations, including those from the US, Europe, and Asia (Japan, China, Korea, etc.) [8,9,10,11,12]. Among them, *GTF2I*, *TP53*, and *NOTCH1* have been identified as key candidates that play critical regulatory roles in the progression of tumors. *GTF2I* encodes a multifunctional General Transcription Factor II-I (TFII-I) that regulates gene expression programs essential for the differentiation and proliferation of thymic epithelial cells and has been linked to the initiation of tumors in indolent thymoma types, with mutation rates reaching approximately 39% [12,13]. The p.L404H (c.1271T>A) mutation has been reported to be unique to TETs and represents the most prevalent genetic alteration in types A (100%) and AB (~70%) [14]. This mutation has been found only in 8% of high-risk thymomas and thymic carcinomas [13,15]. Functionally, this mutation has been implicated in promoting cellular proliferation while exhibiting a limited oncogenic effect, indicating its involvement in the development of tumors with less aggressive malignant transformation [16,17].

*TP53* functions as a central tumor suppressor that regulates DNA repair, cell-cycle arrest, and apoptosis in response to cellular stress [18]. *TP53* was frequently mutated at a lower incidence of roughly 11% [12]. The dysfunction of *TP53* has been reported to result in genomic instability and advancement of malignancy. Mutations in *TP53* are commonly linked to genomic instability and more aggressive subtypes of thymomas (types B2 and B3) as well as thymic carcinomas [16]. Numerous mutations have been documented at hotspot residues, including p.R175, p.R248, and p.R273, which can lead to impaired DNA-binding ability, loss of tumor suppressor functions, and gain-of-function oncogenic properties [16,19]. Another key target gene is *NOTCH1*, which functions as a transmembrane receptor regulating thymocyte development and epithelial cell fate decisions [20]. Although mutations rarely occur in *NOTCH1*, its aberrant activation has been involved in oncogenic signaling and impaired differentiation in metastatic thymomas or thymic carcinoma, in conjunction with *TP53*, *mTOR*, *EGFR*, *CDK4*, and *ASXL1* [16,21].

While foundational studies in North American, European, and other East Asian cohorts have identified key genetic drivers of TETs, the targeted molecular characterization of these tumors in other ethnic groups, such as the Vietnamese population, remains largely unexplored. Furthermore, differentiating high-risk from low-risk TETs based on conventional histopathology can be challenging, creating a need for objective molecular markers to improve risk stratification. A critical knowledge gap therefore exists regarding whether population-specific variants contribute to the disease and could serve as novel biomarkers.

Although thymic epithelial tumors have been genetically investigated in different regions of the world, a comprehensive analysis has not been conducted in the Vietnamese population, primarily due to constraints in sample availability and the small size of cohorts. This situation highlights the necessity for localized molecular data to validate existing findings and explore possible variants specific to the Vietnamese population. This study aimed to characterize the clinicopathological features of a cohort of 150 samples from patients diagnosed with TETs using hematoxylin and eosin (H&E) staining and immunohistochemistry (IHC), and to classify them according to the WHO and Masaoka–Koga systems. Of the 150 samples, 139 were further subjected to targeted Sanger sequencing to identify variants in three key genes, *GTF2I*, *TP53*, and *NOTCH1*. Detected variants were further evaluated for potential pathogenicity using MutationTaster2025 and CADD v1.7, alongside analyses of genotype distribution. The findings of this study will provide valuable insights into the impacts of *GTF2I*, *TP53*, and *NOTCH1* variants, elucidate their genetic associations with thymic epithelial tumor subtypes, and support their potential use as candidate molecular markers for subtype differentiation, although further validation is required. We present the following article in accordance with the STROBE reporting checklist.

## 2. Materials and Methods

### 2.1. Study Design

This was a single-center, cross-sectional study conducted at the Department of Pathology and Forensic Medicine, Military Hospital 103, in Hanoi, Vietnam. A cohort of randomly selected Vietnamese patients was established through both retrospective and prospective collection of archived formalin-fixed, paraffin-embedded (FFPE) tissue samples, which were obtained from tumor resection specimens. A total of 150 patients (aged from 18 to 81) diagnosed with TETs between December 2014 and December 2024 were included. Clinical data were retrieved from corresponding medical records. The sample size was calculated using the formula outlined by Pourhoseingholi [22], with an expected prevalence (P) of 0.1, a 95% confidence level (Z-score), and a 5% margin of error (d).

This study complied with the Declaration of Helsinki. The protocol of this study was reviewed and approved by the ethics committee of Military Hospital 103 with approval No. 353/CNChT—HĐĐĐ. All participants provided written informed consent for the use of their tissue samples and clinical data for research purposes.

### 2.2. Study Population and Sample Selection

The study population consisted of patients with a histopathologically confirmed diagnosis of TET at Military Hospital 103. The inclusion criteria for this study were as follows: (1) diagnosis established between December 2014 and December 2024; (2) availability of a representative FFPE tissue block in the pathology archive; and (3) availability of corresponding clinical and pathological data. Patients were excluded if (1) the tissue block contained insufficient tumor material for molecular analysis or (2) clinical records were incomplete.

A consecutive sampling method was used to include all patients who met these criteria within the study period. From an initial cohort of 150 eligible cases, 11 samples were subsequently excluded from molecular analyses due to poor DNA quality or quantity following extraction. This resulted in a final cohort of 139 samples for genetic analysis.

### 2.3. Tumor Classification and Staging

The diagnosis and tissue classification of TET samples adhered to the guidelines established by the WHO, which divided TET into five histological types: A, AB, B1, B2, and B3 based on their cellular composition and degree of malignancy [2]. Type A is typically considered non-threatening with minimal risk of malignancy, distinguished by its spindle-shaped, epithelial-rich cells and low lymphocyte content. Type AB is a low-grade malignant tumor; it displays a varied composition, integrating areas rich in spindle cells (similar to type A) and regions rich in lymphocytes (similar to type B). Type B1 exhibits a low level of malignancy and resemblance to the normal thymus, characterized by a high presence of immature T-cells. Type B2 shows an elevated potential for malignancy, marked by a greater abundance of epithelial cells and a moderate level of lymphocytes. Type B3 demonstrates a higher degree of malignancy, characterized by a significant quantity of epithelial cells and a limited occurrence of lymphocytes. In contrast, thymic carcinomas, which do not fall under the A or B classifications, exhibit a more aggressive nature and possess distinct molecular characteristics. The TET tissue samples underwent fixation with formalin, followed by embedding in paraffin, and were subsequently stored in the hospital pathology archive at −20 °C.

The staging of TET samples followed the Masaoka staging system, including modifications proposed by Koga [4]. This staging system is employed as the standard classification to assess tumor invasion in Vietnam. It has demonstrated significant relevance and all medical records and surgical reports at the time of data collection were documented according to this scheme. Particularly, Stage I indicates a tumor that is completely encapsulated with no sign of microscopic invasion; Stage II is further divided into IIA and IIB, where IIA shows microscopic invasion into the capsule and IIB reveals macroscopic invasion into surrounding fatty tissue or mediastinal pleura. Stage III demonstrates invasion into neighboring organs, while Stage IV is divided into IVA and IVB, characterized by the dissemination to pleural or pericardial surfaces and lymphatic or distant hematogenous metastases, respectively.

The classification and staging of the TET samples were independently and blindly conducted by two pathologists at the Military Hospital 103 in accordance with the WHO guidelines and the Masaoka–Koga staging system [3,23].

### 2.4. Histological and Immunohistochemical Analyses

The hematoxylin and eosin staining of FFPE TET tissue samples was conducted in accordance with the protocol outlined by Fischer [24]. Briefly, H&E staining was performed on approximately 4 μm TET sections on a slide. Section samples were deparaffinized in xylene and subsequently stained with Mayer’s hematoxylin (0.1%) for 5 min. This was followed by staining with Eosin Y (0.5–1%) for 3 min, resulting in the development of a pinkish-red coloration. The morphology of TET tissue samples was examined under an Olympus BX43F microscope (Olympus, Tokyo, Japan), and pathological diagnosis was established according to the 2015 WHO classification by two independent pathologists [2].

IHC staining was also conducted on 4 μm sections derived from FFPE tissue blocks using monoclonal antibodies: CD45 (MAD-002066QD, cocktail of clones 2B11 and PD7/26, dilution 1:50; RRID: AB_10896120), p53 (MAD-000309QD, clone SP5, dilution 1:50; RRID: AB_653753), EMA (MAD-001100QD, clone E29, dilution 1:100; RRID: AB_1150012) (Invitro Master Diagnostica, Madrid, Spain), and NOTCH1 (CF500078, clone OTI3E12, dilution 1:50; Thermo Fisher Scientific, Waltham, MA, USA; RRID: AB_1547339). All IHC staining was in accordance with the protocol outlined by Bancroft [25]. Before the staining process, tissue sections were mounted on slides, subjected to deparaffinization using xylene, and subsequently rehydrated through a series of graded ethanol solutions (100%, 95%, and 70%). To improve the staining sensitivity and specificity, tissue sections were treated with citrate buffer (pH 6.0) at high temperature (95–100 °C), followed by exposure to 0.3% hydrogen peroxide. Post preparation of tissue samples, the slices were incubated with primary antibodies overnight at 4 °C according to the manufacturer guidelines before applying a combination of biotinylated secondary antibody and streptavidin-conjugated peroxidase (Agilent, Hørsholm, Denmark) for 30 to 60 min at room temperature. For chromogenic detection, a solution of 3,3′-diaminobenzidine (DAB) was used, accompanied by the application of hematoxylin for counterstaining. The change in coloration at the site of the antigen–antibody complex was observed using an Olympus BX43F microscope (Olympus, Tokyo, Japan). The immunoreactivity of CD45, P53, EMA, and NOTCH1 was semi-quantitatively evaluated based on staining intensity scores (0 = negative (no staining or < 1% positive cells), 1+ = weak staining, 2+ = moderate staining, and 3+ = strong staining) and the proportion of positive cells at that staining intensity [26,27]. The histochemical (H) score is calculated as the sum of the proportion of positively stained cells at each intensity level, multiplied by the respective staining intensity score, resulting in an analytical range from 0 to 300 [28].

### 2.5. DNA Extraction and Variant Analysis

Genomic DNA from 139 out of 150 TET patients was successfully extracted from formalin-fixed and paraffin-embedded (FFPE) tumor tissues by the QIAamp DNA FFPE Tissue Kit (Qiagen, Germantown, MD, USA) with some minor modifications. Samples exhibiting low DNA yield or poor DNA quality, along with tissue specimens that lacked adequate tumor cells (such as those with a high presence of normal lymphocytes or epithelial cells), were omitted to prevent any potential bias in subsequent analyses. This sample size remains adequate for conducting molecular and statistical analyses according to the sample size calculation of Pourhoseingholi [22]. The extracted DNA samples were used for genotyping exon 15 of *GTF2I*, exon 7 of *TP53*, and exon 34 of *NOTCH1* using the listed primer sets (Table 1). The exons of *GTF2I*, *TP53*, and *NOTCH1* were selected for variant screening through Sanger sequencing due to their established significance in tumor biology. Exon 15 of GTF2I was a well-established mutation hotspot in thymomas, accounting for more than 95% of all reported *GTF2I* mutations in TETs [29,30]. Exon 7 of *TP53* encodes the DNA-binding domain, with mutations in this region being closely associated with cancer risk and prognosis [29,30]. Exon 34 of *NOTCH1* serves as a mutation hotspot within the PEST domain, a critical region where genetic alterations can disrupt NOTCH1 signaling across various cancers [31,32].

The amplification was carried out in a thermocycler (Eppendorf, Enfield, CT, USA) using the DreamTaq DNA Polymerase (Thermo Fisher Scientific, Waltham, MA, USA). The amplification cycles were set up as follows: (1) an initial denaturation at 96 °C for 10 min, (2) 30 cycles of 96 °C, 58 °C and 72 °C for 30 s each step, and (3) a final extension step at 72 °C for 5 min. The PCR products were purified and sequenced using a 3100 Genetic Analyzer (Applied Biosystems, Foster City, CA, USA) with the BigDye^TM^ Terminator Cycle Sequencing Kit (Thermo Fisher Scientific, Waltham, MA, USA). The obtained sequence data were aligned with the *TP53*, *NOTCH1*, and *GTF2I* reference sequences (Table 1) using the Clustal-Omega v1.2.4 online tool ve1.2.4 (RRID: SCR_001591) with default settings.

Data related to the human *TP53*, *GTF2I*, and *NOTCH1* were retrieved from the NCBI and Ensembl databases. Single-nucleotide polymorphism (SNP) information for these genes was retrieved from the NCBI’s SNP database.

For prediction of possible impacts of genetic variants at DNA and protein levels, *in silico* analysis was conducted using the six different web-based prediction tools, including MutationTaster2025 (RRID: SCR_010777) and CADD v1.7 (Combined Annotation Dependent Depletion; RRID: SCR_018393) [33,34]. Two categories were used to present the prediction outcomes for MutationTaster: harmless (benign) and harmful (deleterious). CADD v1.7 assesses the potential harmfulness of genetic variants through a Phred scoring system (RRID: SCR_001017) that ranges from 1 to 99, with scores greater than 20 indicating a greater probability of the variant causing damage.

Three-dimensional structural analysis was performed using Swiss-PdbViewer (version 4.1.0; RRID: SCR_013295). The protein structure was obtained from the RCSB Protein Data Bank and used for visualization and mutation mapping.

To assess the statistical relationship between variants of *GTF2I*, *TP53*, and *NOTCH1* and the risk of the disease, TET cases were divided into two groups based on both pathological classification and clinical practice. Group 1 (*n* = 78) consisted of types A, AB, and B1, which are generally characterized by less aggressive characteristics, fewer atypical cells, and a more favorable prognosis. In contrast, group 2 (*n* = 61) included types B2 and B3, which exhibit more aggressive histological features, such as increased cellular atypia and higher proliferative activity. This grouping not only highlighted fundamental biological differences but also facilitated the comparison of genetic profiles, haplotypes, and patterns by associating them with tumor characteristics and patient outcomes [2,35].

### 2.6. Analysis of Co-Occurrence Patterns

Co-occurrence patterns among the four statistically significant variants (FDR < 0.05) were analyzed at the individual patient level and visualized using OncoPrint-style heatmaps using Python v3.12.

### 2.7. Statistical Analysis

All statistical analyses were performed using SPSS version 20.0 (IBM Corp., Armonk, NY, USA; RRID: SCR_002865) unless otherwise stated. A two-sided *p*-value < 0.05 was considered statistically significant.

Descriptive statistics were used to summarize patient clinicopathological characteristics. Continuous variables were presented as median and range, while categorical variables were reported as frequencies and percentages (*n*, %).

The primary analysis involved comparing genotype frequencies of the identified variants between the low-risk (Group 1: Types A, AB, B1) and high-risk (Group 2: Types B2, B3) tumor groups. These comparisons were conducted using Fisher’s exact test for all contingency tables. Odds ratios (ORs) and their 95% confidence intervals (CIs) were calculated to estimate the association strength. To account for multiple comparisons across 17 variants, the Benjamini–Hochberg false discovery rate (FDR) correction was applied using Python version 3.12 [36].

## 3. Results

### 3.1. Clinicopathological Characteristics of the Study Cohort

Clinicopathological data from 150 Vietnamese patients diagnosed with TETs are summarized in Table 1 and Table S1. The median age at which individuals received their diagnosis was 51 years, with a range spanning from 18 to 81 years. The demographic distribution revealed that individuals under the age of 50 represented the most significant segment at 48.7%. The 50 to 69 age group accounted for 40.0%, while those aged 70 or above constituted 11.3% of the population. The study cohort exhibited a higher proportion of males (60%), while the females accounted for 40% of the population. Myasthenia gravis (MG), a common paraneoplastic syndrome linked to thymomas, was observed in 92 individuals, accounting for 61.3% of the cases. The majority of patients were diagnosed with MG at early stages I (11 cases), IIA (72 cases), and IIB (7 cases). Only two MG samples were linked to aggressive tumors, comprising one case in stage III and another in stage IVB. The average tumor size measured 49 mm, with a range of 4 to 160 mm, demonstrating significant variation in tumor diameters among the thymic epithelial tumors studied. This difference may reflect unique patterns in tumor growth dynamics or disease progression across instances.

### 3.2. Histological and Immunohistochemical Features of TET Subtypes

In histological classification, thymomas were classified according to the WHO system (Table 2). The most prevalent subtype was type B2, accounting for 29.3%, followed by type AB at 22.0%, B1 at 20.0%, B3 at 14.7%, and type A at 14.0% (Table 2). According to the Masaoka–Koga staging, almost half of the patients (47.4%) were classified as stage I, which indicates the presence of non-invasive encapsulated tumors. The distribution of the remaining patients across progressive stages was as follows: 13.3% were classified in stage IIA, 18.0% in stage IIB, 18.0% in stage III, 1.3% in stage IVA, and 2.0% in stage IVB. The correlation between classification by WHO histologic and clinical staging according to Masaoka–Koga was further demonstrated in Table 3. Less aggressive and lower-grade types (types A, AB, and B1) exhibited a strong correlation with the early Masaoka–Koga stages (I and II). A significant proportion of these tumor types were detected at stage I, specifically, 17 out of 21 samples (80.9%) for type A, 22 out of 33 (66.7%) for type AB, and 20 out of 30 (66.7%) for B1, indicating a predominance of early-stage disease. Conversely, more aggressive and higher-grade types (types B2 and B3) were linked to advanced clinical stages, with 25 samples identified at stage II. Type B3 TET had the highest proportion of advanced disease, with 14 (63.6%) and 3 (13.6%) samples at stages III and IV, respectively.

**Figure 1:**
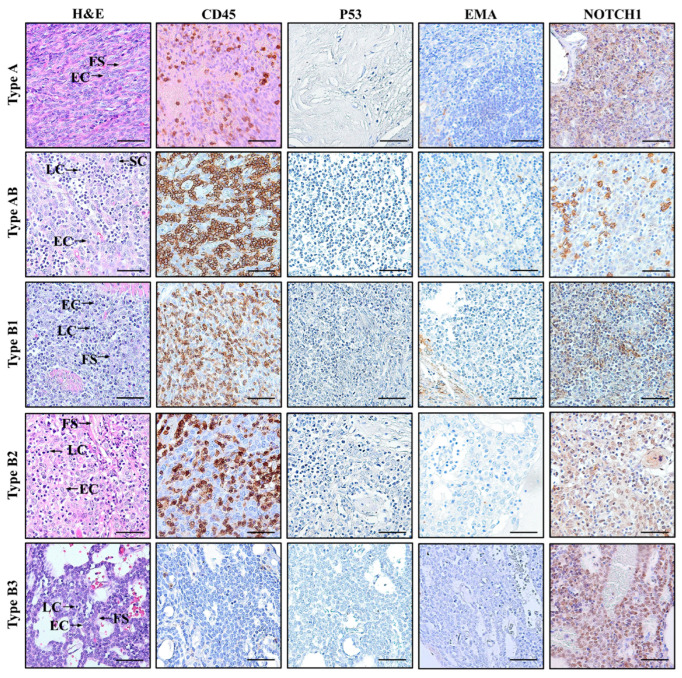
Histopathological and immunohistochemical (IHC) characterization of different tumor types of TETs. H&E staining reveals distinct patterns in types A, AB, B1, B2, and B3 of TETs in accordance with WHO classification. IHC characterization was measured by the immunoreactivity of CD45, p53, EMA, and NOTCH1 antibodies. Images were visualized under an Olympus BX43F microscope at 40× magnification. Bar = 50 µm. FS: fibrous stroma, EC: epithelial cells, LC: lymphocytes, SC: spindle cells.

The histologic features of TET samples exhibited variation across different types (Figure 1). The application of H&E staining revealed a range of histological patterns that are consistent with the distinct type of TET as classified by WHO guidelines [2,37]. In type A, the neoplasm primarily comprised spindle- to oval-shaped epithelial cells. These cells appeared to have finely structured and relatively uniform nuclei, which were embedded in thick fibrous septa. The type AB thymoma section showed two distinct components: lymphocyte-poor and lymphocyte-rich regions. The former region is primarily composed of spindle epithelial cells encapsulated with thick fibrous septa. The latter regions are densely packed with small, mature-appearing lymphocytes, while epithelial cells appear scattered throughout the lymphocytes. In certain instances, these lymphocyte-poor and lymphocyte-rich regions can be densely mixed. The section of type B1 contains a dense mixture of lymphocytes, fibrous septa, and epithelial cells with large nuclei. Among the five TET types, it is observed that the lymphocyte population in type B1 is more abundant compared to the others. The type B2 thymoma section revealed a neoplasm composed of a cluster of epithelial cells exhibiting irregular, hyperchromatic nuclei and coarse chromatin structure. These cells were situated within a thick fibrous stroma, with a limited presence of lymphocytes. The type B3 thymoma section showed sheets of tumor cells with polygonal (round or elongated) epithelial morphology, exhibiting hyperchromatic, grooved, non-uniform nuclei, and dense fibrous stroma. Some necrotic areas were also observed in type B3 TET samples.

IHC analysis revealed strong CD45, EMA, and NOTCH1 expression in all thymoma subtypes, whereas TP53 expression was weak and restricted to advanced types B2–B3 (Figure 1; Table S2). H-scores for these markers demonstrated consistent patterns corresponding to tumor grade. Particularly, CD45, known as leukocyte common antigen (LCA), serves as a useful marker for the identification of immune cells, especially lymphocytes, in TET samples [38]. The expression of CD45 exhibits the highest intensity in type B1 (3+, 80%, H-score = 240) with a gradual decline in types AB = type B3 (H-scores = 150) > type B2 (H-score = 120) > type A (H-score = 90; Figure 1; Table S2).

NOTCH1 was used as a marker in IHC to evaluate the activation level of the NOTCH signaling pathway in TETs (Figure 1). The expression pattern of NOTCH1 is strongly correlated with TET development, with a progressive increase in NOTCH1 expression from type A (3+, 10%, H-score = 30) to type B3 (3+, 80%, H-score 240; Table S2). Staining results revealed that the p53 expression is generally limited to advanced, high-grade types of TETs. Particularly, types B2 and B3 exhibited moderate intensity of p53 staining (2+), with approximately 5% (H-score = 10) and 10% (H-score = 20) of tumor cells, respectively. In contrast, low-grade TET types A, AB and B1 showed an absence of detectable p53 staining.

All thymoma types exhibited clear immunoreactivity for EMA, an epithelial cell-specific marker that highlights the presence and distribution of epithelial components within the tumor (Figure 1). Nevertheless, no clear differences in EMA intensity were detected across all the assessed samples (Table S2).

### 3.3. Spectrum of GTF2I, TP53, and NOTCH1 Variants in TETs

**Figure 2:**
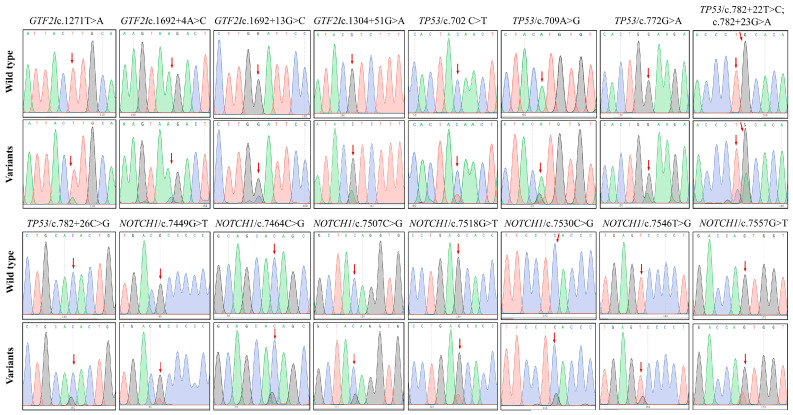
Polymorphism analyses of *GTF2I*, *TP53*, and *NOTCH1* in TET samples. Sanger chromatograms reveal the variant genotypes of *GTF2I* (4 variants), *TP53* (6 variants), and *NOTCH1* (7 variants) in comparison with the wild-type sequence. The red arrows indicate the position of the base changes.

Out of the 150 TET samples collected, 11 were excluded from molecular analyses due to insufficient DNA quality extracted from FFPE samples. High-quality DNA from the remaining 139 TET samples was subjected to variant screening in exons 15, 7, and 34 of three target genes, *GTF2I*, *TP53*, and *NOTCH1*, respectively, using Sanger sequencing. The sequencing data (Figure 2) identified a total of 17 heterozygous variants within the targeted regions, comprising 10 missense variants (58.8%), six intronic variants (35.3%), and one synonymous variant (5.9%) (Table 4).

Particularly, *GTF2I* contains one missense variant (c.1271T>C) and three intronic substitutions (c.1304+51G>A, c.1692+4A>C, and c.1692+13G>C). *TP53* demonstrated the greatest level of variant diversity, including three intronic alterations (c.782+22T>C, c.782+23G>A, and c.782+26C>G), two missense variants (c.709A>G and c.772G>A) and one synonymous variant (c.702C>T). In the *NOTCH1* gene, seven missense variants were identified, including c.7449G>T, c.7464C>G, c.7507C>G, c.7518G>T, c.7530C>G, c.7546T>G, and c.7557G>T. Based on *in silico* predictions, established literature, and statistical associations, the identified variants were classified into the following categories: (a) Known driver/recurrent alteration: *GTF2I*/c.1271T>A (p.L424H), a well-established recurrent driver mutation in thymomas; (b) Previously unreported variants of potential significance: *TP53*/c.772G>A (p.E258K) and *NOTCH1*/c.7546T>G (p.S2516A), both predicted deleterious and statistically enriched in aggressive subtypes; (c) Variants of uncertain significance: intronic variants with statistical associations but predicted benign function (*GTF2I*/c.1304+51G>A); and (d) Likely benign polymorphisms: synonymous and intronic variants with benign predictions (*TP53*/c.702C>T).

**Figure 3:**
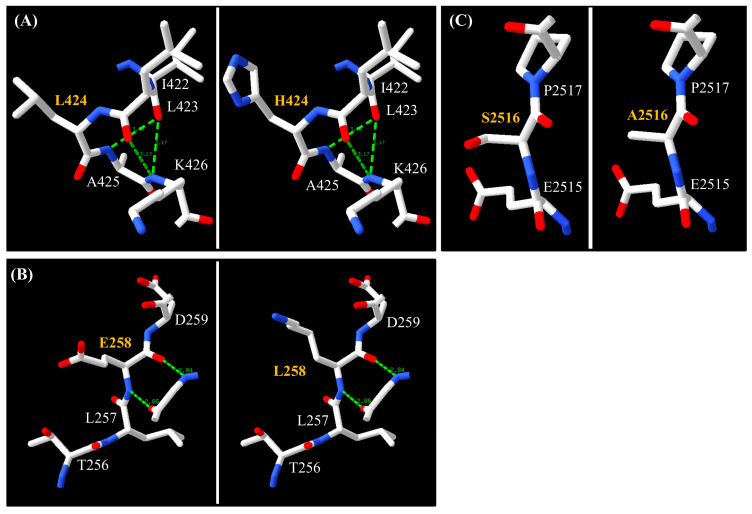
The three-dimensional structure of the *GTF2I* (**A**), *TP53* (**B**) and *NOTCH1* at mutation sites by Swiss-PdbViewer v4.1.0 tool. Left panels represent the reference structures, and right panels represent the corresponding mutated structures (**C**). Blue tip, amide hydrogen; red tip, backbone oxygen; green dashed lines, hydrogen bonds.

To further explore the potential structural impact of selected missense variants, three-dimensional protein models were constructed and visualized using Swiss-PdbViewer v4.1.0. The mutations were found to result in amino acid substitutions with differences in physicochemical properties and side-chain volumes (Figure 3). In GTF2I, the variant p.L424H replaced leucine, a non-polar aliphatic residue with a volume of 166.7 Angstrom^3^ (Å^3^), with histidine, a smaller and polar residue containing an imidazole side chain (153.2 Å^3^), at position 424 (Figure 3A). In TP53, the variant p.E258K resulted in the substitution of glutamic acid, a polar negatively charged amino acid (138.4 Å^3^), by lysine, a larger polar positively charged residue (168.6 Å^3^), at codon 258. The substitution occurs within a region containing T256, L257, and D259 (Figure 3B). In NOTCH1, the variant p.S2516A is located within the C-terminal region corresponding to the PEST domain (Figure 3C). The substitution involved the replacement of serine, a polar uncharged residue (89.0 Å^3^), by a smaller non-polar residue, alanine (88.6 Å^3^), at position 2516.

We next performed the primary analysis to determine whether their frequencies were significantly different between the less aggressive (Group 1) and more aggressive (Group 2) tumor subtypes.

### 3.4. Association of GTF2I, TP53, and NOTCH1 Variants with TET Aggressiveness

We identified specific variants in *GTF2I*, *TP53*, and *NOTCH1* that showed significant differences between Group 1 and Group 2 (Table 5). Two variants, c.1271T>A and c.1304+51G>A, are located in *GTF2I*. The TA genotype of the c.1271T>A variant was detected in 14.1% of patients in Group 1 but was not observed in Group 2 (0.0%). This distribution indicates a statistically significant correlation with the less aggressive Group 1, supported by an odds ratio (OR) of 0.048, a 95% confidence interval (CI) of 0.003–0.827 and an adjusted *p*-value of 0.014 (Table 5). The minor allele frequency (MAF) for this variant in Group 1 was measured at 0.071 (Table 4). Conversely, the c.1304+51G>A variant demonstrated an opposing pattern, with the AG genotype showing a higher frequency in Group 2 (18.0%) compared to Group 1 (0.0%). This intronic variant was associated with an increased risk of TET (OR: 35.7, 95% CI: 2.06–620.2, and an adjusted *p*-value = 0.0011; Table 5), with a low minor allele frequency (MAF = 0.09; Table 4). The third variant, c.772G>A, is situated in *TP53.* The prevalence of this variant was markedly higher in Group 2 (16.4%) compared with Group 1 (1.3%), yielding a high OR of 15.098, a 95% CI spanning from 1.875 to 121.567 and an adjusted *p*-value of 0.01 (Table 5). Furthermore, c.772G>A is infrequently observed within the population, with MAF = 0.006 in Group 1 and 0.082 in Group 2. The fourth variant of interest was *NOTCH1*/c.7546T>G. The TG genotype of c.7546T>G was detected in 9.8% of patients in Group 2 and was absent in Group 1. This distribution corresponded to an OR of 18.387, with a 95% CI ranging from 1.015 to 333.160, and an adjusted *p*-value = 0.026 (Table 5), and a MAF value of 0.049 (Table 4). These data suggest that there is a notable correlation between the c.7546T>G variant with the more aggressive tumor subtype.

### 3.5. Co-Occurrence Patterns Among Key Risk-Associated Variants

**Figure 4:**
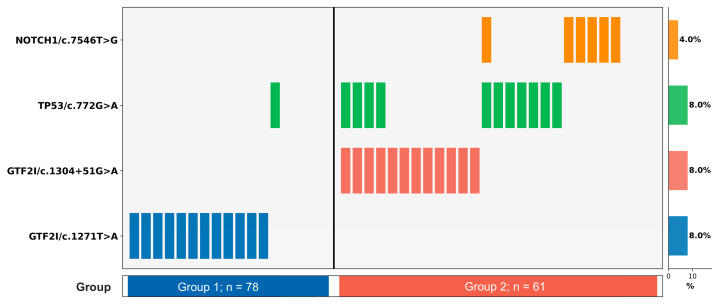
Distribution of four variants across Group 1 (types A, AB, B1; *n* = 78) and Group 2 (types B2, B3; *n* = 61) in TET. Each column represents one sample, and each row represents one variant. The right panel shows the overall frequency of each variant across all samples (%).

An exploratory analysis of sample distribution and co-occurrence patterns was performed on the four variants that showed statistically significant differences between groups (*GTF2I*/c.1271T>A, *GTF2I*/c.1304+51G>A, *TP53*/c.772G>A, and *NOTCH1*/c.7546T>G). The *GTF2I*/c.1271T>A was exclusively detected in Group 1, while *TP53*/c.772G>A and *NOTCH1*/c.7546T>G were predominantly found in Group 2 (Figure 4). These patterns suggest a degree of mutual between indolent-associated and aggressive-associated variants, although the limited sample size precludes definitive conclusions about co-occurrence or mutual exclusivity. Importantly, these observations reflect tumor-level variant patterns and should not be interpreted as population-level genetic linkage, as the variants reside on different chromosomes (*GTF2I* on chromosome 7, *TP53* on chromosome 17, and *NOTCH1* on chromosome 9). Pairwise co-occurrence analysis was performed using Fisher’s exact test. A significant co-occurrence was observed between *GTF2I*/c.1304+51G>A and *TP53*/c.772G>A (expected co-occurrence = 0.96, OR = 8.125, *p*-value = 0.0084), indicating a non-random pattern of co-occurrence. In contrast, no other variation combinations were statistically significant.

## 4. Discussion

Thymic epithelial tumors are primary neoplasms located in the anterior mediastinum, characterized by a wide range of histological subtypes and clinical behaviors [1]. Despite their rarity, they are clinically important because of diagnostic complexity and treatment difficulties. Differentiating high-risk from low-risk TETs remains challenging due to their histologic and clinical heterogeneity [39,40]. Addressing this diagnostic gap, our study investigates molecular markers that strongly correlate with tumor aggressiveness in a Vietnamese patient cohort. We demonstrate that the *GTF2I*/c.1271T>A variant is strongly associated with indolent thymomas (Group 1), whereas previously unreported potentially deleterious variants in *TP53* (c.772G>A) and *NOTCH1* (c.7546T>G) are predominantly observed in more aggressive subtypes (Group 2). These findings suggest that incorporating these genetic markers alongside traditional classification may improve risk stratification and patient management. In the following sections, we will first discuss the prevalence and protective association of the *GTF2I* variant in indolent thymomas within the context of existing literature. Subsequently, we will explore the potential pathogenic roles of the novel risk-associated variants in *TP53* and *NOTCH1* in more aggressive TETs, and conclude with the study’s limitations and future directions. Finally, we will address the study’s limitations and propose future directions based on these results.

In the present study, we collected and classified a cohort of 150 TET samples according to WHO and Masaoka–Koga criteria, and summarized their clinicopathological features [2,3]. It is worth noting that this is one of the largest TET cohorts that has been investigated to date in Vietnam. Our study demonstrates a clear correlation between histologic aggressiveness and clinical invasiveness in TETs (Table 1 and Table 2). Specifically, tumors classified as lower grade and exhibiting less aggressive histology (types A, AB, and B1) were primarily observed in early-stage disease (stages I and II), whereas higher-grade subtypes B2 and B3 were typically associated with more advanced stages (stages III and IV) and more severe conditions [5,6]. A large proportion of patients in our cohort have also been found to develop MG, an autoimmune neuromuscular disorder [41], accounting for 61.3% of the Vietnamese patient population. This observation is in line with previous reports, where MG has been documented in up to 55% of thymoma patients [42]. The slightly higher prevalence in our study may reflect population-specific characteristics, and further highlighting this thymomas-MG association. Building on this well-characterized clinicopathological foundation, we next performed variant screening and molecular analysis to explore potential genetic associations with TET subtypes.

Molecular analysis of 139 TET samples revealed significant genetic alterations. High-quality DNA was subjected to variant screening in exons 15, 7, and 34 of three target genes, *GTF2I*, *TP53*, and *NOTCH1*, respectively, employing Sanger sequencing technique. The selection of these exons was based on their representation of hotspot regions characterized by a high frequency of genetic alterations in TETs. Exon 15 of *GTF2I* is where most documented mutations are found, with earlier investigations suggesting mutation rates of over 40% [11,43]. For *TP53*, variants were exclusively identified within exon 7, whereas other frequently mutated exons (exons 5 and 8) showed no genetic alterations [19,44]. Exon 34 of *NOTCH1*, which encodes the PEST domain, was selected because it is susceptible to gain-of-function mutations [45,46]. Focusing on these exons enabled efficient and scalable detection of functionally relevant variants across a large cohort, offering a practical approach to investigate potential genetic associations of three target genes with each TET subtype.

Two variants in *GTF2I* were identified in our cohort that exhibited intergroup differences. The c.1271T>A variant affects the second TFII-I repeat adjacent to the DNA-binding interface, substituting a hydrophobic leucine with a positively charged histidine (p.L424H) [43,47,48]. This structural modification has a deleterious effect on the TFII-I conformation and DNA-binding capacity, thereby promoting TET development via dysregulation of transcriptional regulation [13,43,49]. Our *in silico* prediction results also indicate a high likelihood of functional impact from the c.1271T>A variant, with MutationTaster indicating a deleterious effect and a CADD score of 25.6 (Table S3). The c.1271T>A (p.L424H) variant in GTF2I exhibits a high frequency in thymoma types A (82%) and AB (74%), and it was first discovered by Petrini (*n* = 127 patients) [29]. The frequency of this variant in our Vietnamese cohort (14.1% in Group 1) is lower than reported in Japanese (63.6–100%, *n* = 31 individuals; 64%, *n* = 22 individuals), Chinese (17–70%, *n* = 296 individuals), India (21.6–56.8%, *n* = 37 individuals) and German (19.5–40.3%, *n* = 77 individuals) cohorts, which may reflect genuine population-specific differences in genetic background, environmental exposures, or partially result from differences in detection methodology and cohort composition [8,13,43,50,51]. This variant was completely absent in subtypes with greater aggressiveness, Group 2 (0%). Statistical analysis further supported the association of the c.1271T>A variant with Group 1, with an OR of 0.048 (95% CI: 0.003–0.827, adjusted *p*-value = 0.014), suggesting that this minor allele may confer a protective effect against the development of more aggressive tumor subtypes. The observed MAF of 0.071 suggests that the c.1271T>A variant is relatively common within the Vietnamese population of Group 1. In contrast, while the intronic c.1304+51G>A variant was present in 18.0% of Group 2 patients, yielding an OR of 35.753 (95% CI: 2.061–620.226, adjusted *p*-value = 0.0011), yet *in silico* analyses predicted it to be functionally benign. This result indicates that, despite its evident association with the Group 2 population, this variant may not have a direct functional role in the progression of the disease.

Mutations in *TP53* represent one of the most frequently observed genetic alterations in TETs, with their occurrence varying across histological subtypes [52]. In general, *TP53* mutations are infrequently detected in indolent thymomas (types A, AB, and B1), with reported frequencies between 3% and 14%. However, these mutations are considerably more prevalent (approximately 30%) in aggressive thymomas and thymic carcinomas [1,10]. In various tumor types, mutations in *TP53* have been consistently linked to unfavorable prognosis, including increased rates of recurrence and reduced rates of overall survival [52,53]. In the present study, the *TP53* mutation rate was observed in 16.4% of patients in Group 2, consistent with TCGA and other large cohorts reporting rates of 20–30% [21]. This finding supports the association of the *TP53* variant with disease progression and histological severity. In contrast, merely 1.3% of Group 1 patients carried the *TP53* variant, consistent with previously reported low frequencies in indolent thymomas. Comparative analysis between the two groups revealed a markedly increased OR of 15.098 (95% CI: 1.875–121.567, adjusted *p*-value = 0.01) for the previously unreported c.772G>A variant. This variant is more prevalent in Group 2 (MAF: 0.082), indicating an increase of over 13 times in the minor allele frequency compared to Group 1 [54]. In addition, the high OR emphasizes its strong association with Group 2; however, the wide confidence interval reveals statistical uncertainty and suggests that larger cohorts are required to enhance risk assessments regarding the variant, the disease progression, and histological severity. Moreover, the functional impact of the c.772G>A variant was computationally predicted using MutationTaster2025 and CADD v1.7 tools. The results provided additional evidence for the pathogenic potential of this variant, with MutationTaster classifying it as deleterious and CADD giving a score of 44 (Table S3). The p.E258K substitution occurs within loop L3 of the highly conserved DNA-binding domain of TP53, a region directly involved in DNA contact. This mutation introduces a charge inversion at codon 258, replacing a negatively charged glutamic acid with a positively charged lysine, which is predicted to disrupt critical electrostatic interactions. As a result, protein stability and DNA binding capacity may be impaired. These effects are similar to those observed in well-characterized pathogenic mutations at neighboring residues (e.g., p.R248W, p.R273H) [30]. Functionally, this disruption is expected to impair transcriptional activation of key downstream targets, including p21 (cell-cycle arrest), BAX and PUMA (apoptosis), and GADD45 (DNA repair), thereby compromising the TP53-mediated tumor suppression pathway. This pathway-level dysregulation is consistent with the aggressive phenotype observed in Group 2 tumors harboring this variant. Similar missense mutations within this domain (e.g., p.L145Q, p.V157F, p.R282W, p.G154V, p.R158P, and p.R273C) have been demonstrated to promote tumorigenesis in TETs by affecting the p53 DNA-binding capacity and disrupting transcriptional regulation of target genes, leading to either loss- or gain-of-function oncogenic activities [19,44].

*NOTCH1* is not a commonly mutated gene in TETs, and its genetic alterations have been reported to be associated with aggressive thymomas and thymic carcinomas [16,21]. At the molecular level, gain-of-function mutations in *NOTCH1* exhibit oncogenic properties across various cancer types [45,55], while loss-of-function alterations have been shown to disrupt the Wnt/β-catenin signaling pathway and cell differentiation in laryngeal cancer [56,57]. In our cohort, the *NOTCH1* variant (c.7546T>G, p.S2516A) was identified as a previously unreported variant in the regulatory PEST domain, characterized by a high concentration of proline (P), glutamic acid (E), serine (S), and threonine (T) [31,32,45]. This variant is located near residues S2513, S2523, and Q2487 within the PEST domain. Mutations at these sites (p.S2513A, p.S2523L, and p.Q2487L) have been experimentally validated to enhance NOTCH1 intracellular domain (NICD) stability, resulting in its accumulation, sustained signaling, and increased oncogenic activity [45,46]. Considering this closeness, the p.S2516A substitution might equally disrupt NICD degradation and amplify oncogenic signaling in TETs. At the pathway level, stabilized NICD would sustain activation of downstream NOTCH target genes (e.g., *HES1*, *HEY1*, *MYC*), promoting cell proliferation and survival while inhibiting differentiation. Furthermore, aberrant NOTCH signaling has been shown to crosstalk with other oncogenic pathways including Wnt/beta-catenin, mTOR, and NF-kappaB, potentially creating a network of sustained pro-tumorigenic signals in aggressive TETs. Consistent with this interpretation, *in silico* analyses by MutationTaster2025 and CADD v1.7 tools predicted that the variant is likely deleterious (Table S3). Furthermore, it was a relatively low-frequency variant (MAF = 0.049) detected in 9.8% of Group 2 patients, showing a strong statistical association with disease progression (OR: 18.387; 95% CI: 1.015–333.16, adjusted *p*-value = 0.026). Although the wide confidence interval indicates a degree of statistical uncertainty, this markedly elevated OR implies a potential association between this *NOTCH1* variant and disease progression in aggressive TET subtypes within the Vietnamese population.

Several limitations of this study should be acknowledged. First, as only tumor FFPE tissue was analyzed without matched normal tissue, we cannot definitively classify the detected variants as somatic or germline. While the *GTF2I*/c.1271T>A variant has been extensively characterized as a somatic event in prior studies using paired tumor-normal sequencing [12,29], the somatic versus germline origin of the previously unreported *TP53*/c.772G>A and *NOTCH1*/c.7546T>G variants cannot be confirmed without matched normal tissue analysis. Their absence from major population databases (e.g., gnomAD) supports but does not confirm a somatic origin. Future studies should incorporate matched tumor-normal sequencing to resolve this question. First, the relatively small sample size, particularly when analyzing individual variants within subgroups, resulted in wide confidence intervals for some risk estimates, such as those for the *TP53* and *NOTCH1* variants. This statistical uncertainty underscores that our findings, while significant, should be interpreted with caution until validated in larger cohorts. Second, as a single-center study, our findings may not be fully generalizable to the broader Vietnamese population or other ethnic groups with different genetic backgrounds and environmental exposures. Third, our analysis was restricted to specific exons of *GTF2I*, *TP53*, and *NOTCH1*. This targeted approach, while effective for known hotspots, may have missed other potentially pathogenic variants. Specifically, well-known *TP53* hotspot mutations at codons R175 (exon 5), R248, and R273 (exon 8) were not surveyed, and we estimate our approach captures approximately 30–40% of total TP53 mutations in TETs. Similarly, *NOTCH1* mutations in the HD domain (exons 26–27) were not assessed. Nonetheless, this targeted strategy provides a cost-effective and scalable approach suitable for resource-limited settings, focusing on the most functionally relevant and well-characterized regions. This approach may have missed other potentially pathogenic variants located in other exonic or regulatory regions of these genes, or in other genes relevant to TET pathogenesis. From a translational perspective, these findings may have implications for personalized management of TETs. The *GTF2I*/c.1271T>A variant, as a marker of indolent behavior, could support conservative management strategies and potentially spare patients from unnecessary aggressive treatment. Conversely, TP53 variant status may inform eligibility for p53-reactivating agents (e.g., APR-246/eprenetapopt) or immunotherapy, as TP53-mutant tumors often exhibit higher tumor mutational burden. NOTCH1 PEST domain variants may identify patients who could benefit from gamma-secretase inhibitor therapy, which has shown preclinical efficacy in cancers with similar NOTCH1 alterations [47]. Future studies should aim to integrate histological classification, targeted genetic screening, IHC biomarker panels, and transcriptomic/immune profiling data into a composite molecular risk model for TETs. Despite these limitations, our study provides the first targeted molecular characterization of these key genes in a well-defined Vietnamese TET cohort, laying a crucial foundation for future, larger-scale investigations.

## 5. Conclusions

Our study within a Vietnamese population demonstrates distinct associations of three genetic variants, *GTF2I*/c.1271T>A (p.L424H), *TP53*/c.772G>A (p.E258K), and *NOTCH1*/c.7546T>G (p.S2516A), with different thymoma subtypes. These findings provide novel insights into the targeted molecular characterization of thymic epithelial tumors in the Vietnamese population. The *GTF2I* variant was strongly linked to indolent thymomas, while the *TP53* and *NOTCH1* variants were associated with more aggressive subtypes of TETs. Exploratory co-occurrence analysis revealed patterns of mutual exclusivity between indolent-associated (*GTF2I*) and aggressive-associated (*TP53*, *NOTCH1*) variants at the tumor level. These findings provide hypothesis-generating evidence suggesting that a combined assessment of these three variants could potentially serve as a complementary molecular tool for distinguishing TET subtypes. However, these associations require functional validation and confirmation in larger, independent cohorts with longitudinal follow-up before any clinical significance can be established. However, due to the limited sample size for each variant within the assessed population, additional validation in larger and independent cohorts is required.

## Figures and Tables

**Table 1:** List of primers used for genotyping of TET samples.

Gene	Ensembl ID	Primers (5′–3′)	Amplicon (bp)
*TP53*	ENSG00000141510	F: CAGGTCTCCCCAAGGCGCAC R: GCAAGCAGAGGCTGGGGCAC	219
*NOTCH1*	ENSG00000148400	F: TGCACACTATTCTGCCCCAG R: ACTTGAAGGCCTCCGGAATG	309
*GTF2I*	ENSG00000263001	F: AAGCCAAAGGTCCGGTGAC R: ACATAGAACCTAGTGGTGAATGAAT	268

**Table 2:** Clinicopathological characteristics of the study population.

Variable	Total (*n* = 150)
Age, years	
Median [range]	51 [18–81]
<50	73 (48.7%)
50–69	60 (40.0%)
≥70	17 (11.3%)
Sex	
Male	90 (60.0%)
Female	60 (40.0%)
Tumor size, largest dimension (mm)	
Median [range]	49 [4–160]
WHO histologic classification	
Type A	21 (14.0%)
Type AB	33 (22.0%)
Type B1	30 (20.0%)
Type B2	44 (29.3%)
Type B3	22 (14.7%)
Masaoka–Koga stage	
Stage I	71 (47.4%)
Stage IIA	20 (13.3%)
Stage IIB	27 (18.0%)
Stage III	27 (18.0%)
Stage IVA	2 (1.3%)
Stage IVB	3 (2.0%)
Clinical diagnosis	
Myasthenia gravis	92 (61.3%)
Stage I	11
Stage IIA	72
Stage IIB	7
Stage III	1
Stage IVA	0
Stage IVB	1
Others	58 (38.7%)

Data are presented as n (%) for categorical variables and median [range] for continuous variables. WHO, World Health Organization.

**Table 3:** WHO histologic classification of TETs distributed according to Masaoka–Koga clinical stages (*n* = 150).

	Stage I	Stage II	Stage III	Stage IV	Total
**Type A**	17	2	2	0	21
**Type AB**	22	8	2	1	33
**Type B1**	20	8	2	0	30
**Type B2**	11	25	7	1	44
**Type B3**	1	4	14	3	22
**Total**	71	47	27	5	150

**Table 4:** General information on variants of *GTF2I*, *TP53*, and *NOTCH1* genes in TET types in Group 1 (types A, AB, and B1) and Group 2 (types B2 and B3).

Gene/Variant	Protein Change	Functional Consequences	*In Silico* Prediction	MAF *		*p*-Value		
				Group 1	Group 2	Group 1	Group 2	All Population
*GTF2I*/c.1271T>A	**L424H**	Missense	Deleterious	0.071	NaN	0.799	NaN	0.887
*GTF2I*/c.1304+51G>A	**-**	Intron	Benign	NaN **	0.090	NaN	0.741	0.887
*GTF2I*/c.1692+4A>C	**-**	Intron	Deleterious	0.026	NaN	0.973	NaN	0.985
*GTF2I*/c.1692+13G>C	**-**	Intron	Benign	0.026	NaN	0.973	NaN	0.985
*TP53*/c.702C>T	N235N	Synonymous	Benign	0.006	0.041	0.998	0.946	0.967
*TP53*/c.709A>G	M237V	Missense	Deleterious	0.013	NaN	0.993	NaN	0.996
*TP53*/c.772G>A	**E258K**	Missense	Deleterious	0.006	0.082	0.998	0.784	0.887
*TP53*/c.782+22T>C	**-**	Intron	Benign	0.006	0.016	0.998	0.992	0.992
*TP53*/c.782+23G>A	**-**	Intron	Benign	0.218	0.172	0.048	0.268	0.015
*TP53*/c.782+26C>G	**-**	Intron	Benign	0.013	0.000	0.993	NaN	0.996
*NOTCH1*/c.7449G>T	T2483M	Missense	Benign	0.032	0.016	0.958	0.992	0.955
*NOTCH1*/c.7464C>G	H2488Q	Missense	Benign	0.013	0.033	0.993	0.966	0.967
*NOTCH1*/c.7507C>G	Q2503E	Missense	Deleterious	0.032	0.082	0.958	0.784	0.798
*NOTCH1*/c.7518G>T	E2506D	Missense	Benign	0.077	0.131	0.763	0.499	0.418
*NOTCH1*/c.7530C>G	T2471S	Missense	Deleterious	0.045	0.090	0.918	0.741	0.717
*NOTCH1*/c.7546T>G	**S2516A**	Missense	Deleterious	NaN	0.049	NaN	0.922	0.967
*NOTCH1*/c.7557G>T	Q2519H	Missense	Benign	0.064	0.016	0.833	0.992	0.868

* MAF (Minor Allele Frequency) values were calculated for each subgroup (All Population, *n* = 139) and within each subgroup. A *p*-value < 0.05 indicates a statistically significant association. ** NaN (not a number) indicates that the MAF could not be calculated because the variant was not observed in that subgroup. The bold format is to highlight the key variants.

**Table 5:** Comparison of genotype frequencies of *GTF2I*, *TP53*, and *NOTCH1* genes in Group 1 (types A, AB, and B1; *n* = 78) and Group 2 (types B2 and B3; *n* = 61).

Gene/Variant	Test Model	Group 1 (Frequency)	Group 2 (Frequency)	OR *	95% CI *	*p*-Value *	FDR (Adj *p*-Value)
*GTF2I*/c.1271T>A	TT	67 (85.9%)	61 (100%)	1			
	TA	11 (14.1%)	0 (0.0%)	0.048	0.003 to 0.827	**0.0024**	**0.014**
*GTF2I*/c.1304+51G>A	GG	78 (100%)	50 (82.0%)	1			
	GA	0 (0%)	11 (18.0%)	35.753	2.061 to 620.226	**0.0001**	**0.0011**
*GTF2I*/c.1692+4A>C	AA	74 (94.9%)	61 (100%)	1			
	AC	4 (5.1%)	0 (0.0%)	0.135	0.007 to 2.549	0.131	0.213
*GTF2I*/c.1692+13G>C	GG	74 (94.9%)	61 (100%)	1			
	GC	4 (5.1%)	0 (0.0%)	0.135	0.007 to 2.549	0.131	0.213
*TP53*/c.702C>T	CC	77 (98.7%)	56 (91.8%)	1			
	CT	1 (1.3%)	5 (8.2%)	6.875	0.782 to 60.484	0.086	0.213
*TP53*/c.709A>G	AA	76 (97.4%)	61 (100%)	1			
	AG	2 (2.6%)	0 (0.0%)	0.249	0.012 to 5.279	0.504	0.535
*TP53*/c.772G>A	GG	77 (98.7%)	51 (83.6%)	1			
	GA	1 (1.3%)	10 (16.4%)	15.098	1.875 to 121.567	**0.0012**	**0.010**
*TP53*/c.782+22T>C	TT	77 (98.7%)	59 (96.7%)				
	TC	1 (1.3%)	2 (3.3%)	2.610	0.231 to 29.481	0.582	0.582
*TP53*/c.782+23G>A	GG	44 (56.4%)	40 (65.6%)	1			
	GA	34 (43.6%)	21 (34.4%)	0.679	0.340 to 1.358	0.298	0.423
*TP53*/c.782+26C>G	CC	76 (97.4%)	61 (100%)	1			
	CG	2 (2.6%)	0 (0.0%)	0.249	0.012 to 5.279	0.504	0.535
*NOTCH1*/c.7449G>T	GG	73 (93.6%)	59 (96.7%)	1			
	GT	5 (6.4%)	2 (3.3%)	0.495	0.093 to 2.643	0.466	0.535
*NOTCH1*/c.7464C>G	CC	76 (97.4%)	57 (93.4%)	1			
	CG	2 (2.6%)	4 (6.6%)	2.667	0.472 to 15.069	0.404	0.529
*NOTCH1*/c.7507C>G	CC	73(93.6%)	51 (83.6%)	1			
	CG	5 (6.4%)	10 (16.4%)	2.863	0.923 to 8.876	0.096	0.213
*NOTCH1*/c.7518G>T	GG	66 (84.6%)	45 (73.8%)	1			
	GT	12 (15.4%)	16 (26.2%)	1.956	0.845 to 4.525	0.138	0.213
*NOTCH1*/c.7530C>G	CC	71 (91.0%)	50 (82.0%)	1			
	CG	7 (9.0%)	11 (18.0%)	2.231	0.809 to 6.154	0.132	0.213
*NOTCH1*/c.7546T>G	TT	78 (100%)	55 (90.2%)	1			
	TG	0 (0.0%)	6 (9.8%)	18.387	1.015 to 333.160	**0.0062**	**0.026**
*NOTCH1*/c.7557G>T	GG	68 (87.2%)	59 (96.7%)	1			
	GT	10 (12.8%)	2 (3.3%)	0.231	0.049 to 1.095	0.067	0.213

* The *p*-values were calculated using Fisher’s exact test and adjusted using false discovery rate Benjamini–Hochberg (FDR; adjusted *p*-value) correction method. A *p*-value < 0.05 indicates a statistically significant association. OR: odds ratio; 95% CI: confidence interval of the odds ratio.

## Data Availability

The original contributions presented in this study are included in the article and Supplementary Material. Further inquiries can be directed to the corresponding author.

## Supplementary Materials

The following supporting information can be downloaded at: https://www.mdpi.com/article/10.3390/genes17050524/s1. Table S1. Clinicopathological characteristics of study population. Table S2. Immunohistochemical findings in TET samples. Table S3. In silico prediction of genetic variants in *GTF2I*, *TP53*, and *NOTCH1* with associated amino acid changes and pathogenicity scores.

## Abbreviations

The following abbreviations are used in this manuscript:

TETs	Thymic Epithelial Tumors
H&E	Hematoxylin and Eosin
IHC	Immunohistochemistry
WHO	World Health Organization
PPFE	Formalin-Fixed, Paraffin-Embedded
SNP	Single-Nucleotide Polymorphism
CADD	Combined Annotation Dependent Depletion
MG	Myasthenia Gravis

## References

[1] Xu S., Li X., Zhang H., Zu L., Yang L., Shi T., Zhu S., Lei X., Song Z., Chen J. (2021). Frequent genetic alterations and their clinical significance in patients with thymic epithelial tumors. Front. Oncol..

[2] Marx A., Chan J.K.C., Chalabreysse L., Dacic S., Detterbeck F., French C.A., Hornick J.L., Inagaki H., Jain D., Lazar A.J. (2022). The 2021 WHO classification of tumors of the thymus and mediastinum: What is new in thymic epithelial, germ cell, and mesenchymal tumors?. J. Thorac. Oncol..

[3] Masaoka A., Monden Y., Nakahara K., Tanioka T. (1981). Follow-up study of thymomas with special reference to their clinical stages. Cancer.

[4] Chiappetta M., Lococo F., Pogliani L., Sperduti I., Tabacco D., Bria E., D’Argento E., Massaccesi M., Boldrini L., Meacci E. (2021). Masaoka-Koga and TNM staging system in thymic epithelial tumors: Prognostic comparison and the role of the number of involved structures. Cancers.

[5] Yoshida Y., Yanagawa M., Sato Y., Miyata T., Kawata A., Hata A., Tomiyama N. (2024). Differential diagnosis between low-risk and high-risk thymoma: Comparison of diagnostic performance of radiologists with and without deep learning model. Acta Radiol. Open.

[6] Liu J., Yin P., Wang S., Liu T., Sun C., Hong N. (2021). CT-based radiomics signatures for predicting the risk categorization of thymic epithelial tumors. Front. Oncol..

[7] Sun X., Wang M., Li X., Yang F., Zhang L. (2023). Incidence of thymic malignancies in China: A longitudinal study based on a national commercial claims database from 2007 to 2016. Chin. Med. J..

[8] Shimada M., Taniguchi H., Yamaguchi H., Gyotoku H., Sasaki D., Kaku N., Senju C., Senju H., Imamura E., Takemoto S. (2023). Genetic profile of thymic epithelial tumors in the Japanese population: An exploratory study examining potential therapeutic targets. Transl. Lung Cancer Res..

[9] De Jong W.K., Blaauwgeers J.L., Schaapveld M., Timens W., Klinkenberg T.J., Groen H.J. (2008). Thymic epithelial tumours: A population-based study of the incidence, diagnostic procedures and therapy. Eur. J. Cancer.

[10] Yang J., Zhang B., Guan W., Fan Z., Pu X., Zhao L., Jiang W., Cai W., Quan X., Miao S. (2023). Molecular genetic characteristics of thymic epithelial tumors with distinct histological subtypes. Cancer Med..

[11] Wang X., Jin H., Feng X., Liang Z., Jin R., Li X. (2024). Depiction of the genetic alterations and molecular landscapes of thymic epithelial tumors: A systematic review and meta-analysis. Cancers.

[12] Radovich M., Pickering C.R., Felau I., Ha G., Zhang H., Jo H., Hoadley K.A., Anur P., Zhang J., McLellan M. (2018). The integrated genomic landscape of thymic epithelial tumors. Cancer Cell.

[13] Feng Y., Lei Y., Wu X., Huang Y., Rao H., Zhang Y., Wang F. (2017). GTF2I mutation frequently occurs in more indolent thymic epithelial tumors and predicts better prognosis. Lung Cancer.

[14] Möhrmann L., Rostock L., Werner M., Oleś M., Arnold J.S., Paramasivam N., Jöhrens K., Rupp L., Schmitz M., Richter D. (2025). Genomic landscape and molecularly informed therapy in thymic carcinoma and other advanced thymic epithelial tumors. Med.

[15] Liu W., Yang H.S., Zheng S.Y., Weng J.H., Luo H.H., Lei Y.Y., Feng Y.F. (2022). Thymic epithelial tumors: Examining the *GTF2I* mutation and developing a novel prognostic signature with LncRNA pairs to predict tumor recurrence. BMC Genom..

[16] Oberndorfer F., Müllauer L. (2020). Genomic alterations in thymoma-molecular pathogenesis?. J. Thorac. Dis..

[17] Manti P.G., Trattaro S., Castaldi D., Pezzali M., Spaggiari L., Testa G. (2022). Thymic stroma and TFII-I: Towards new targeted therapies. Trends Mol. Med..

[18] Aubrey B.J., Strasser A., Kelly G.L. (2016). Tumor-suppressor functions of the TP53 pathway. Cold Spring Harb. Perspect. Med..

[19] Szpechcinski A., Szolkowska M., Winiarski S., Lechowicz U., Wisniewski P., Knetki-Wroblewska M. (2022). Targeted next-generation sequencing of thymic epithelial tumours revealed pathogenic variants in *KIT*, *ERBB2*, *KRAS*, and *TP53* in 30% of thymic carcinomas. Cancers.

[20] Deftos M.L., Huang E., Ojala E.W., Forbush K.A., Bevan M.J. (2000). Notch1 signaling promotes the maturation of CD4 and CD8 SP thymocytes. Immunity.

[21] Ardeshir-Larijani F., Schneider B.P., Althouse S.K., Radovich M., Masood A., Perna F., Salman H., Loehrer P.J. (2023). Clinicogenomic landscape of metastatic thymic epithelial tumors. JCO Precis. Oncol..

[22] Pourhoseingholi M.A., Vahedi M., Rahimzadeh M. (2013). Sample size calculation in medical studies. Gastroenterol. Hepatol. Bed Bench.

[23] Leisibach P., Schneiter D., Soltermann A., Yamada Y., Weder W., Jungraithmayr W. (2016). Prognostic value of immunohistochemical markers in malignant thymic epithelial tumors. J. Thorac. Dis..

[24] Fischer A.H., Jacobson K.A., Rose J., Zeller R. (2008). Hematoxylin and eosin staining of tissue and cell sections. Cold Spring Harb. Protoc..

[25] Bancroft J.D., Gamble M. (2018). Theory and Practice of Histological Techniques.

[26] Gal A.A., Sheppard M.N., Nolen J.D.L., Cohen C. (2004). p53, cellular proliferation, and apoptosis-related factors in thymic neuroendocrine tumors. Mod. Pathol..

[27] Ku X., Sun Q., Zhu L., Gu Z., Han Y., Xu N., Meng C., Yang X., Yan W., Fang W. (2020). Deciphering tissue-based proteome signatures revealed novel subtyping and prognostic markers for thymic epithelial tumors. Mol. Oncol..

[28] Price P., Ganugapati U., Gatalica Z., Kakadekar A., Macpherson J., Quenneville L., Rees H., Slodkowska E., Suresh J., Yu D. (2023). Reinventing nuclear histo-score utilizing inherent morphologic cutoffs: Blue-brown color H-score (BBC-HS). Appl. Immunohistochem. Mol. Morphol..

[29] Piaskowski S., Zawlik I., Szybka M., Kulczycka-Wojdala D., Stoczynska-Fidelus E., Bienkowski M., Robak T., Kusinska R., Jesionek-Kupnicka D., Kordek R. (2010). Detection of P53 mutations in different cancer types is improved by cDNA sequencing. Oncol. Lett..

[30] Hálková T., Ptáčková R., Semyakina A., Suchánek Š., Traboulsi E., Ngo O., Hejcmanová K., Májek O., Bureš J., Zavoral M. (2022). Somatic mutations in cxon 7 of the *TP53* gene in index colorectal lesions are associated with the early occurrence of metachronous adenoma. Cancers.

[31] Edelmann J. (2022). NOTCH1 signalling: A key pathway for the development of high-risk chronic lymphocytic leukaemia. Front. Oncol..

[32] Jelloul F.Z., Yang R., Garces S., Kanagal-Shamanna R., Ok C.Y., Loghavi S., Routbort M.J., Zuo Z., Yin C.C., Floyd K. (2022). Landscape of *NOTCH1* mutations and co-occurring biomarker alterations in chronic lymphocytic leukemia. Leuk. Res..

[33] Schwarz J.M., Cooper D.N., Schuelke M., Seelow D. (2014). MutationTaster2: Mutation prediction for the deep-sequencing age. Nat. Methods.

[34] Schubach M., Maass T., Nazaretyan L., Röner S., Kircher M. (2024). CADD v1.7: Using protein language models, regulatory CNNs and other nucleotide-level scores to improve genome-wide variant predictions. Nucleic Acids Res..

[35] Grajkowska W., Matyja E., Kunicki J., Szymanska S., Marx A., Weis C.A., Langfort R., Szolkowska M. (2017). AB thymoma with atypical type A component with delayed multiple lung and brain metastases. J. Thorac. Dis..

[36] Haynes W., Dubitzky W., Wolkenhauer O., Cho K.-H., Yokota H. (2013). Benjamini–Hochberg method. Encyclopedia of Systems Biology.

[37] von der Thüsen J. (2024). Thymic epithelial tumours: Histopathological classification and differential diagnosis. Histopathology.

[38] Chen Y., Klingen T.A., Aas H., Wik E., Akslen L.A. (2021). Tumor-associated lymphocytes and macrophages are related to stromal elastosis and vascular invasion in breast cancer. J. Pathol. Clin. Res..

[39] Gao C., Yang L., Xu Y., Wang T., Ding H., Gao X., Li L. (2024). Differentiating low-risk thymomas from high-risk thymomas: Preoperative radiomics nomogram based on contrast enhanced CT to minimize unnecessary invasive thoracotomy. BMC Med. Imaging.

[40] Willner J., Zhou F., Moreira A.L. (2022). Diagnostic challenges in the cytology of thymic epithelial neoplasms. Cancers.

[41] Meriggioli M.N., Sanders D.B. (2009). Autoimmune myasthenia gravis: Emerging clinical and biological heterogeneity. Lancet. Neurol..

[42] Bernard C., Frih H., Pasquet F., Kerever S., Jamilloux Y., Tronc F., Guibert B., Isaac S., Devouassoux M., Chalabreysse L. (2016). Thymoma associated with autoimmune diseases: 85 cases and literature review. Autoimmun. Rev..

[43] Petrini I., Meltzer P.S., Kim I.-K., Lucchi M., Park K.-S., Fontanini G., Gao J., Zucali P.A., Calabrese F., Favaretto A. (2014). A specific missense mutation in *GTF2I* occurs at high frequency in thymic epithelial tumors. Nat. Genet..

[44] Calhoun S., Daggett V. (2011). Structural effects of the L145Q, V157F, and R282W cancer-associated mutations in the p53 DNA-binding core domain. Biochemistry.

[45] Wang K., Zhang Q., Li D., Ching K., Zhang C., Zheng X., Ozeck M., Shi S., Li X., Wang H. (2015). PEST domain mutations in Notch receptors comprise an oncogenic driver segment in triple-negative breast cancer sensitive to a γ-secretase inhibitor. Clin. Cancer Res..

[46] Meijer H.A., Hetherington A., Johnson S.J., Gallagher R.L., Hussein I.N., Weng Y., Rae J.M., Noordzij T., Kalamara M., Macartney T.J. (2025). NOTCH1 S2513 is critical for the regulation of NICD levels impacting the segmentation clock in hiPSC-derived PSM cells and somitoids. Genes Dev..

[47] Hsieh M.-S., Kao H.-L., Huang W.-C., Wang S.-Y., Lin S.-Y., Chu P.-Y., Pan C.-C., Chou T.-Y., Ho H.-L., Yeh Y.-C. (2023). Constant p.L424H mutation in *GTF2I* in micronodular thymomas with lymphoid stroma: Evidence supporting close relationship with type A and AB thymomas. Mod. Pathol..

[48] Gurumurthy A., Wu Q., Nar R., Paulsen K., Trumbull A., Fishman R.C., Brand M., Strouboulis J., Qian Z., Bungert J. (2020). TFII-I/*GTF1I* and erythro-Megakaryopoiesis. Front. Physiol..

[49] Kim I.-K., Rao G., Zhao X., Fan R., Avantaggiati M.L., Wang Y., Zhang Y.-W., Giaccone G. (2020). Mutant GTF2I induces cell transformation and metabolic alterations in thymic epithelial cells. Cell Death Differ..

[50] Lang M., Kazdal D., Mohr I., Anamaterou C. (2023). Differences and similarities of *GTF2I* mutated thymomas in different Eurasian ethnic groups. Transl. Lung Cancer Res..

[51] Higuchi R., Goto T., Hirotsu Y., Yokoyama Y., Nakagomi T., Otake S., Amemiya K., Oyama T., Mochizuki H., Omata M. (2020). Primary driver mutations in *GTF2I* specific to the development of thymomas. Cancers.

[52] Moreira A.L., Won H.H., McMillan R., Huang J., Riely G.J., Ladanyi M., Berger M.F. (2015). Massively parallel sequencing identifies recurrent mutations in *TP53* in thymic carcinoma associated with poor prognosis. J. Thorac. Oncol..

[53] Takata S. (2024). Genomic insights into molecular profiling of thymic carcinoma: A narrative review. Mediastinum.

[54] Chattopadhyay A., Lu T.P. (2020). Overcoming the challenges of imputation of rare variants in a Taiwanese cohort. Transl. Cancer Res..

[55] Tosello V., Ferrando A.A. (2013). The NOTCH signaling pathway: Role in the pathogenesis of T-cell acute lymphoblastic leukemia and implication for therapy. Ther. Adv. Hematol..

[56] Gong X.Y., Chen H.B., Zhang L.Q., Chen D.S., Li W., Chen D.H., Xu J., Zhou H., Zhao L.L., Song Y.J. (2022). NOTCH1 mutation associates with impaired immune response and decreased relapse-free survival in patients with resected T1-2N0 laryngeal cancer. Front. Immunol..

[57] D’Assoro A.B., Leon-Ferre R., Braune E.-B., Lendahl U. (2022). Roles of NOTCH signaling in the tumor microenvironment. Int. J. Mol. Sci..

