# Supplementary material for: Distinct Associations of GTF2I, TP53, and NOTCH1 Variants with Indolent and Aggressive Thymic Epithelial Tumors in Vietnamese Patients
Source: Genes (Basel). 2026 Apr 29;17(5):524. doi: 10.3390/genes17050524 (PMC13205841; doi:10.3390/genes17050524)
Supplement: Supplementary file 1 [file genes-17-00524-s001.zip › genes-4266571-supplementary.pdf]

Table S1. Clinicopathological characteristics of study population.

Case no.	Age	Sex	Tumor size (L × W × T; mm)	Clinical diagnosis	WHO histologic classification	Masaoka-Koga stage
1	43	F	8 × 1 × 1	Anemia	AB	IIa
2	44	M	30 ×10 × 1	MG stage IIA, Ptosis, Dysphagia, Dyspnea	AB	I
3	42	M	70 × 65 × 55	Cough	B3	III
4	46	F	43 × 19 × 62	MG stage IIA, Ptosis, Dyspnea	B2	III
5	68	F	24 × 23 × 25	MG stage I, Ptosis	A	I
6	22	F	30 × 16 × 25	MG stage IIA, Paresis	B2	IIb
7	62	M	50 × 40 × 50	Dyspnea	B2	III
8	64	F	27 × 24 × 1	MG stage IIA, Ptosis, Dysarthria, Dysphagia	B2	III
9	41	M	38 × 32 × 40	MG stage IIA, Ptosis, Dysphagia	AB	IIa
10	45	M	28 × 21× 1	MG stage IIA, Ptosis, Dysphagia	AB	IIa
11	53	M	42 × 25 × 57	MG stage IIA, Ptosis, Paresis	B2	IIa
12	43	M	41 × 35 × 40	MG stage IIA, Ptosis	B1	IIb
13	53	F	44 × 33 × 15	MG stage IIA, Ptosis, Dysarthria, Dysphagia	AB	I
14	53	F	22 × 18 × 14	MG stage IIA, Dyspnea	AB	I
15	44	F	69 × 54 × 28	MG stage IIA, Ptosis, Dysarthria, Dysphagia	B2	IIa
16	38	F	36 × 26 × 51	MG stage IIA, Dyspnea	A	I
17	55	M	35 × 25 × 61	MG stage IIA	AB	I
18	21	F	74 × 70 × 60	Fever	B2	IIb
19	56	M	18 × 16 × 1	MG stage IIA, Dysphagia, Dysarthria	A	I
20	44	F	30 × 17 × 43	MG stage IIA, Dyspnea, Fatigue	B1	I
21	50	F	35 × 15 × 1	MG stage IIA, Ptosis	AB	I
22	73	M	20 × 10 × 25	MG stage IIA	AB	I
23	53	M	36 × 31 × 1	MG stage IIA, Paresis	B2	IIa
24	55	M	20 × 12 × 43	MG stage IIA, Ptosis	B2	IIa
25	47	M	47 × 32 × 1	MG stage IIA, Dysphagia, Dysarthria	B1	I
26	54	M	43 × 29 × 50	MG stage IIA, Fatigue	B2	IIa
27	47	F	16 × 12 × 1	MG stage IIA, Ptosis	AB	I

28	64	F	39 × 27 × 1	MG stage IIA, Ptosis, Dysarthria	B2	IIa
29	73	M	41 × 28 × 25	Urinary retention	B2	I
30	56	M	32 × 30 × 1	MG stage IIA, Dysarthria, Dysphagia	B2	I
31	30	M	44 × 7 × 1	MG stage IIA, Dyspnea, Paresis	AB	I
32	59	F	4 × 3 × 1	MG stage I, Ptosis	B1	I
33	73	M	51 × 20 × 43	MG stage IIA, Ptosis	B2	IIa
34	59	M	68 × 65 × 1	Physical examination	A	I
35	20	F	40 × 20 × 35	MG stage I, Ptosis	B2	I
36	44	M	36 × 32 × 1	MG stage IIA, Ptosis, Paresis	AB	I
37	60	M	24 × 10 × 1	MG stage IIA, Dysarthria, Dysphagia	A	I
38	77	M	45 × 40 × 1	Cough, Chest pain	A	I
39	65	F	57 × 37 × 1	Cough, Dyspnea	B1	I
40	65	M	21 × 14 × 20	MG stage IIA, Ptosis	A	I
41	31	M	4 × 3 × 1	MG stage IIA, Ptosis	B2	IIa
42	27	M	37 × 27 × 47	MG stage IIA, Ptosis, Paresis	B2	IIa
43	48	M	21 × 14 × 20	MG stage IIA, Dyspnea	B1	I
44	64	M	48 × 28 × 1	MG stage I, Ptosis	B2	I
45	64	M	24 × 14 × 1	MG stage IIA, Ptosis	AB	IIb
46	32	F	32 × 18 × 1	MG stage IIA, Ptosis	B1	I
47	71	F	63 × 29 × 1	MG stage IIA, Ptosis, Dysarthria	B1	I
48	73	M	50 × 45 × 55	MG stage IIA, Dysarthria	B3	IIb
49	45	F	46 × 21 × 1	MG stage IIA, Ptosis, Dysarthria	B2	IIb
50	28	M	69 × 35 × 51	MG stage IIA	B1	I
51	41	M	26 × 20 × 1	MG stage I, Ptosis	AB	I
52	37	M	6 × 5 × 1	MG stage IIA	B1	IIb
53	53	M	50 × 30 × 1	MG stage I, Ptosis	B2	I
54	49	M	40 × 21 × 36	MG stage I, Dry cough	B2	I
55	31	F	57 × 56 × 90	MG stage IIA	B1	III
56	70	M	67 × 48 × 63	Chest pain	B1	IIb
57	47	F	33 × 28 × 55	MG stage I, Ptosis	A	I

58	30	F	23 × 13 × 30	MG stage IIA, Ptosis	B3	IIb
59	51	M	32 × 16 × 1	MG stage IIA, Ptosis	B2	I
60	66	M	81 × 70 × 84	MG stage IIA	B2	I
61	27	F	52 × 48 × 16	MG stage IIB	B2	I
62	30	M	48 × 20 × 1	MG stage IIA, Ptosis, Paresis	B3	III
63	58	M	80 × 70 × 75	Malnutrition	B3	III
64	54	F	70 × 70 × 50	Chest pain	B3	III
65	79	M	105 × 74 × 1	Physical examination	B3	III
66	78	M	70 × 55 × 1	MG stage IIA, Fatigue	AB	I
67	50	M	40 × 37 × 44	Chest pain	B1	IIb
68	42	F	64 × 50 × 1	MG stage IIA	B2	IIb
69	47	F	44 × 39 × 1	Chest pain	A	I
70	53	F	31 × 13 × 1	MG stage IIA, Ptosis, Paresis	AB	I
71	48	M	61 × 16 × 65	MG stage IIA, Ptosis, Paresis	AB	I
72	57	F	69 × 42 × 1	Chest pain, Cough	B1	I
73	71	M	60 × 40 × 1	Dry cough	A	IIa
74	74	M	40 × 35 × 50	MG stage IIA	B1	I
75	19	M	75 × 70 × 65	MG stage IIA, Paresis	B2	IIa
76	69	M	96 × 88 × 60	Dry cough	A	I
77	40	F	25 × 15 × 27	MG stage IIA	AB	I
78	62	M	12 × 9 × 1	MG stage IIB	AB	I
79	40	M	41 × 35 × 46	MG stage IIA	B1	IIa
80	35	F	36 × 26 × 50	MG stage IIA	A	I
81	26	M	80 × 60 × 40	Pleural effusion	AB	III
82	65	F	60 × 55 × 65	Cough, Chest pain	A	I
83	57	F	55 × 1 × 1	Cough, Dyspnea	B2	I
84	40	F	50 × 15 × 64	MG stage IIA	B2	IIb
85	37	F	50 × 55 × 70	MG stage IIA	B1	I
86	21	F	55 × 20 × 1	MG stage I, Ptosis	B1	I
87	71	M	35 × 10 × 65	Dry cough	A	I

88	56	M	69 × 52 × 75	Chest pain	B1	I
89	26	F	60 × 1 × 1	Dry cough	B1	I
90	49	M	90 × 40 × 1	MG stage IIA	B2	IIa
91	58	F	32 × 23 × 1	MG stage IIA, Ptosis, Dysarthria	A	IIa
92	54	F	34 × 26 × 30	MG stage IVB	B2	III
93	42	F	50 × 18 × 35	MG stage IIB, Ptosis	B2	III
94	71	M	150 × 1 × 1	Chest pain	AB	I
95	42	M	30 × 22 × 1	MG stage IIB, Fatigue	B1	I
96	61	M	66 × 56 × 54	Cough, Chest pain	B3	III
97	65	M	75 × 74 × 64	Hemoptysis	B3	III
98	68	M	86 × 76 × 1	Cough, Chest pain	B1	III
99	69	F	43 × 30 × 55	Chest pain	AB	I
100	62	F	48 × 25 × 33	MG stage IIB	AB	IIb
101	36	F	23 × 18 × 1	MG stage III	B1	I
102	19	F	73 × 60 × 42	Facial edema	B3	IVb
103	18	M	70 × 54 × 67	Physical examination	B3	III
104	53	F	57 × 33 × 60	Physical examination	B1	I
105	58	F	30 × 20 × 20	Physical examination	B1	I
106	40	M	52 × 41 × 30	Physical examination	B2	III
107	49	F	7 × 1 × 1	Physical examination	AB	I
108	33	M	30 × 25 × 1	MG stage IIA, Ptosis	B2	IIb
109	70	M	74 × 62 × 109	Cough, Chest pain	B2	IIb
110	26	M	150 × 120 × 90	Pleural effusion	AB	IVA
111	56	M	120 × 104 × 105	Physical examination	A	I
112	44	M	56 × 52 × 45	Cough, Chest pain	B2	IVa
113	41	M	70 × 40 × 20	Physical examination	AB	I
114	67	M	67 × 50 × 70	Cough, Chest pain	AB	I
115	41	M	39 × 19 × 39	MG stage IIB, Dyspnea	B3	I
116	21	M	75 × 50 × 1	Physical examination	B2	III
117	32	F	30 × 10 × 1	Physical examination	B3	IVb

118	21	M	134 × 75 × 97	Respiratory failure	A	III
119	33	M	50 × 41 × 1	Dyspnea	B1	I
120	62	F	88 × 70 × 50	MG stage IIB	B3	IVb
121	44	F	60 × 45 × 1	MG stage IIA	B2	IIa
122	52	M	95 × 85 × 4	Chest pain	B3	III
123	35	F	90 × 70 × 50	Chest pain	A	III
124	49	M	50 × 35 × 1	MG stage IIA	B1	IIa
125	45	F	65 × 40 × 30	MG stage IIA	B2	IIa
126	63	M	35 × 20 × 4	Hypertension	AB	IIb
127	58	F	100 × 90 × 50	Dyspnea	AB	IIb
128	73	M	30 × 30 × 1	Cough	AB	IIb
129	39	M	160 × 120 × 1	Chest pain	AB	I
130	51	M	60 × 30 × 1	Cough	A	I
131	62	M	10 × 10 × 1	MG stage IIA	B2	I
132	52	M	50 × 30 × 20	MG stage IIA	B2	IIb
133	69	M	80 × 60 × 40	Physical examination	AB	III
134	35	F	90 × 70 × 50	Physical examination	A	I
135	52	M	95 × 85 × 4	Mediastinal tumor	B3	III
136	58	F	90 × 60 × 40	MG stage IIA	B2	IIb
137	46	F	35 × 20 × 10	MG stage IIA	B3	IIb
138	60	M	25 × 20 × 40	Mediastinal tumor	B3	IIb
139	58	M	30 × 20 × 35	Mediastinal tumor	B2	IIb
140	19	M	55 × 40 × 30	MG stage IIA	B3	III
141	64	F	60 × 50 × 43	MG stage IIA	B2	IIb
142	81	F	60 × 35 × 48	Mediastinal tumor	A	I
143	49	F	63 × 50 × 32	Mediastinal tumor	B1	IIb
144	56	M	52 × 50 × 32	MG stage IIA	B3	III
145	53	M	50 × 32 × 60	MG stage IIA	B3	III
146	24	M	23 × 18 × 3	MG stage I	B1	I
147	41	M	56 × 24 × 35	MG stage IIA	B2	IIb

148	26	F	40 × 32 × 36	MG stage IIA	B1	IIb
149	25	M	74 × 64 × 120	MG stage IIA	B3	III
150	71	M	50 × 30 × 40	MG stage I	AB	I

Table S2. Immunohistochemical findings in TET samples

No.	Types	CD45	H-score	P53	H-score	EMA	H-score	NOTCH1	H-score
1	A	3+ 30%	90	0	0	3+ 5%	15	3+ 10%	30
2	AB	3+ 50%	150	0	0	3+ 5%	15	3+ 10%	30
3	B1	3+ 80%	240	0	0	3+ 15%	45	3+ 70%	210
4	B2	3+ 40%	120	2+ 5%	10	3+ 10%	30	3+ 50%	150
5	B3	3+ 50%	150	2+ 10%	20	3+ 5%	15	3+ 80%	240

Note: 0 to 3+: IHC staining intensity with 0 = no staining, 1+ = weak staining, 2+ = moderate staining and 3+ = strong staining. Percentage values: proportion of tumor cells that are positive for the marker at that staining intensity [1,2].

References

1. Gal AA, Sheppard MN, Nolen JDL, Cohen C: p53, cellular proliferation, and apoptosis-related factors in thymic neuroendocrine tumors. Mod Pathol 17(1): 33-39, 2004.
2. Ku X, Sun Q, Zhu L, Gu Z, Han Y, Xu N, Meng C, Yang X, Yan W, *et al.*: Deciphering tissue-based proteome signatures revealed novel subtyping and prognostic markers for thymic epithelial tumors. Mol Oncol 14(4): 721-741, 2020.

Table S3. *In silico* prediction of genetic variants in *GTF2I*, *TP53*, and *NOTCH1* with associated amino acid changes and pathogenicity scores.

Genes	Variants	Position on GRCh37	Position on GRCh38	Aa change	*In silico* prediction	
					Mutant Taster	CADD
GTF2I/ NM_032999	c.1271T>A	Chr7:74197053	Chr7:74732629	**L424H**	Deleterious	25.6
	c.1304+51G>A	Chr7:74147054	Chr7:74732713	Intron	Benign	1.7
	c.1692+4A>C	Chr7:74147007	Chr7:74732666	Intron	Deleterious	22.9
	c.1692+13G>C	Chr7:74147016	Chr7:74732675	Intron	Benign	7.7
TP53/ NM_00546	c.702C>T	Chr17:7577579	Chr17:7674261	N235N	Benign	0.6
	c.709A>G	Chr17:7577572	Chr17:7674254	M237V	Deleterious	25.8
	c.772G>A	Chr17:7577509	Chr17:7674191	**E258K**	Deleterious	44
	c.782+22T>C	Chr17:7577477	Chr17:7674159	Intron	Benign	8.9
	c.782+23G>A	Chr17:7577476	Chr17:7674158	Intron	Benign	1.7
	c.782+26C>G	Chr17:7577473	Chr17:7674155	Intron	Benign	5.2
NOTCH1/ NM_017617	c.7449G>T	Chr9:139390742	Chr9:136496290	T2483M	Benign	6.2
	c.7464C>G	Chr9:139390727	Chr9:136496275	H2488Q	Benign	5.4
	c.7507C>G	Chr9:139390684	Chr9:136496232	Q2503E	Deleterious	48
	c.7518G>T	Chr9:139390673	Chr9:136496221	E2506D	Benign	8.9
	c.7530C>G	Chr9:139390659	Chr9:136496207	T2471S	Deleterious	27
	c.7546T>G	Chr9:139390645	Chr9:136496193	**S2516A**	Deleterious	26.5
	c.7557G>T	Chr9:139390634	Chr9:136496182	Q2519H	Benign	8.9
